# Leveraging academic initiatives to advance implementation practice: a scoping review of capacity building interventions

**DOI:** 10.1186/s13012-022-01216-5

**Published:** 2022-07-23

**Authors:** Lisa A. Juckett, Alicia C. Bunger, Molly M. McNett, Monica L. Robinson, Sharon J. Tucker

**Affiliations:** 1 School of Health and Rehabilitation Sciences, Division of Occupational Therapy, The Ohio State University, Columbus, USA; 2 College of Social Work, The Ohio State University, Columbus, USA; 3 College of Nursing, Helene Fuld Health Trust National Institute for Evidence-Based Practice in Nursing & Healthcare, The Ohio State University, Columbus, USA

**Keywords:** Implementation capacity building interventions, Implementation practice, Academic institutions

## Abstract

**Background:**

Academic institutions building capacity for implementation scholarship are also well positioned to build capacity in real world health and human service settings. How practitioners and policy makers are included and trained in implementation capacity-building initiatives, and their impact on building implementation practice capacity is unclear. This scoping review identified and examined features of interventions that build implementation practice capacity across researchers and practitioners or practitioners-in-training.

**Methods:**

Five bibliographic databases were searched. Eligible studies (a) described an implementation capacity building intervention with a connection to an academic institution, (b) targeted researchers and practitioners (including practitioners-in-training, students, or educators), and (c) reported intervention or participant outcomes. Articles that only described capacity building interventions without reporting outcomes were excluded. Consistent with Arksey and O’Malley’s framework, key study characteristics were extracted (target participants, core components, and outcomes) and analyzed using open coding and numerical analysis.

**Results:**

Of 1349 studies identified, 64 met eligibility for full-text review, and 14 were included in the final analysis. Half of the studies described implementation capacity building interventions that targeted health or behavioral health researchers, practitioners, and practitioners-in-training together, and half targeted practitioners or practitioners-in-training only. The most common components included structured didactic activities offered in person or online, mentorship and expert consultation to support implementation, and practical application activities (e.g., field placements, case studies). Knowledge sharing activities and technical assistance were less common. All studies reported favorable outcomes related to knowledge attainment, increased ability to implement evidence, productivity, and satisfaction.

**Conclusions:**

Building implementation capacity among practitioners is critical for integrating insights from implementation science into the field and preventing the “secondary” implementation research-to-practice gap. This scoping review identified several promising implementation practice capacity building interventions that tend to build practitioner capacity via expert led activities which may be relevant for academic institutions seeking to build implementation practice capacity. To avoid widening the implementation research-to-practice gap, implementation capacity building interventions are needed that target policy makers, expand beyond multiple practice settings, and leverage university/community partnerships or on-site academic medical centers. Future studies will also be needed to test the impact on service quality and public health outcomes.

**Supplementary Information:**

The online version contains supplementary material available at 10.1186/s13012-022-01216-5.

Contributions to the literature

- Implementation practice capacity building interventions are needed for integrating insights from implementation science into practice; however, they have received less attention than initiatives focused on building implementation research capacity.
- This scoping review identified 14 implementation capacity building interventions that included practitioners or trainees. Results demonstrated how interventions have targeted a range of stakeholders including practice leaders and students, often using traditional didactic or mentored training approaches with a goal of improving implementation knowledge, application, confidence, and productivity.
- Implementation capacity building interventions that target policy makers and multiple practice settings are also needed.

## Introduction

The exponential and continued growth of the field of implementation science underscores its relevance, importance, and scientific value in bridging the research to practice gap. The adoption of research evidence into routine practice remains complex and influenced by a number of organizational, system, and individual factors [1]. Advances in implementation science have identified and validated strategies using a consistent nomenclature to facilitate the implementation process [2, 3]. Similarly, development of specific theories, models, and frameworks that draw on multiple disciplinary traditions provide a systematic approach to implementation and stimulate interdisciplinary teams of researchers and practitioners that can break down silo-based work to promote and sustain implementation efforts [4, 5]. This team-based approach to implementation requires that practitioners and researchers share resources and a commitment to an evidence-based approach to implementation. Prior reviews of implementation capacity building interventions describe academic training opportunities in implementation and acknowledge a need for greater engagement and attention to implementation practitioners and policy makers [6, 7]. Indeed, building implementation capacity among professionals who are tasked with implementing evidence in routine practice and policy settings is critical for carrying out implementation studies and scaling the benefits of evidence-based interventions. As such, greater understanding is needed for how academic institutions might design or refine their efforts to build capacity among researchers *and* practitioners—the frontline professionals who are tasked with implementing evidence in routine care.

Across both academic and practice settings, the process of capacity building is ladened with unique challenges. First, implementation science is a rapidly evolving field, making it difficult for capacity building initiatives to reflect the field’s most current discoveries and advancements [8, 9]. Second, the demand for capacity building interventions, including implementation-focused trainings, didactic activities, and workshops, exceeds the availability [10]. Third, well-established capacity building interventions have primarily targeted the implementation research community, resulting a major knowledge gap for how best to build implementation capacity among the practitioners (e.g., nurses, social workers, physicians, therapists, pharmacists) who are responsible for applying evidence in daily practice. Programs such as the Training Institute for Dissemination and Implementation Research in Health [11, 12], the Implementation Research Institute [13], and the Mentored Training for Dissemination and Implementation Research in Cancer [14] are historically comprised of researchers with activities geared towards grantsmanship and mentorship from expert academicians.

Implementation researchers are interested in generating knowledge regarding the processes, strategies and methods that promote uptake of evidence-based interventions by clinicians, organizations, systems, and patients. Implementation practitioners are users of implementation science to accelerate the adoption and application of evidence-based interventions. Importantly, implementation researchers and practitioners are not always mutually exclusive groups, as many actively engage in both generation and integration of the science. Even when capacity building interventions are designed for a multi-stakeholder audience, it remains difficult to tailor such interventions to the unique needs of all attendees given their heterogeneities in implementation experiences, trajectories, and professional backgrounds [12]. These challenges do not represent an exhaustive list but rather depict the complexities of developing, deploying, and sustaining effective capacity building interventions for implementation stakeholders. It is critically important to engage multiple stakeholders at the practice level to prevent development of a “secondary gap,” where the knowledge from implementation science is not applied among front-line teams seeking to implement evidence-based practices in real-world settings [15, 16].

In an effort to advance implementation capacity, implementation leaders have recently encouraged the development of interventions that build capacity across a diverse group of individual learners and multidisciplinary teams [7, 17]. In particular, Davis and D’Lima’s [7] recent systematic review on implementation research capacity building interventions underscored the critical need to include practitioners, pre-doctoral trainees, and policymakers, especially those in low resource settings with limited access to financial supports and personnel. Indeed, building implementation capacity among researchers without also building capacity among those on the ground who are responsible for implementing new practices potentially widens the divide between implementation research and implementation practice [15, 18]—an ironic gap with potential to restrict the extent to which implementation theories, models, frameworks, and strategies are used in both clinical and community contexts [16]. While the demand for research-based implementation capacity building interventions will continue to grow in academic settings, these interventions should be deliberately designed to also build practice capacity. Doing so will foster collaborations among researchers, practitioners, and trainees, leading to productive partnerships and the development of multidisciplinary implementation networks that span research and practice settings.

Though the concept of building capacity in a multi-contextual manner may garner favorable attention from both researchers and practitioners, little is known about effective interventions that can build implementation practice capacities across these groups of stakeholders simultaneously. Accordingly, the present scoping review aimed to examine interventions and programs designed to build implementation practice capacity in the following formats: (a) collectively among researchers, practitioners, and/or students; (b) among practitioners, and (c) among practitioners-in-training (e.g., graduate students). Identifying these interventions, as well as their core components and outcomes, is a crucial precursor to deploying interventions that effectively build the implementation practice capacity of diverse groups of learners. Addressing practice capacity narrows the gap between implementation science and implementation practice by efficiently moving evidence-based interventions into practice to improve healthcare safety and quality.

## Methods

Our scoping review protocol (available upon request) was established a priori and aligned with Arskey and O’Malley’s [19] five-stage framework for review methodology. A scoping review approach was selected given the nascent nature of capacity building interventions in implementation science and, unlike systematic reviews, the scoping review methodology allowed the review team to assess the breadth and depth of the capacity building literature through an iterative search process [20]. The review was conducted by a team with expertise in implementation science, implementation practice, and scoping review methodology. The Preferred Reporting Items for Systematic Reviews and Meta-Analysis, Scoping Review Extension (PRISMA-ScR) was used for organization and presentation of key findings (Additional file 1) [21].

### Stage 1: Identifying the research question

Our team of implementation researchers, consisting of researchers and practitioners in the fields of rehabilitation, social work, and nursing, collaborated to develop the current review’s research question: *What interventions (e.g., courses, mentorship, workshops) have been used to build implementation capacity among researchers, practitioners, and/or students?* Our secondary focus was to assess the types of outcomes measured to determine capacity building intervention effectiveness.

### Stage 2: Identifying relevant studies

**Fig. 1:**
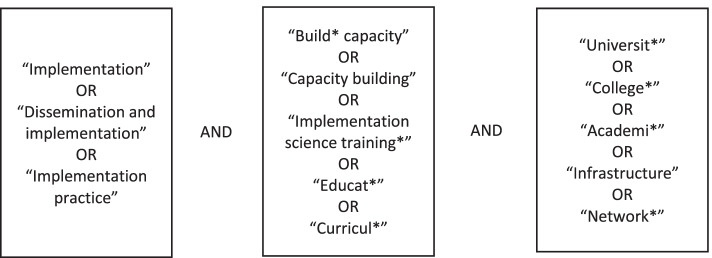
Search terms for identifying relevant studies

To identify relevant studies, five electronic bibliographic databases were accessed using a librarian assisted search strategy (Academic Search Complete, CINAHL, MEDLINE, PsycINFO, and SocINDEX). We entered search terms that represented dissemination and implementation, capacity building, and academic institutions given that many capacity building interventions have been developed using academic resources. For instance, the following string of search terms was used to search our five databases simultaneously: [“implementation”] AND [“build* capacity”] AND [“university” OR “college” OR “higher education”]. Our full list of search terms can be found in Fig. 1. All eligible studies that were identified through our search strategy were uploaded into the web-based scoping and systematic review program, Covidence [22], prior to initiating stage 3.

### Stage 3: Study selection

Study selection consisted of a two-step process. In step 1, two members of the review team with experience in scoping review methodology and implementation research (LAJ and MLR) screened all eligible titles and abstracts. Team members applied the inclusion and exclusion criteria listed below.

#### Inclusion criteria:

- Studies that examined capacity building interventions across researchers, practitioners, and practitioners-in-training:
  - Examples of interventions included didactic courses, workshops/trainings, mentoring and expert consultation, practical application activities, knowledge sharing activities, and project support
  - Studies were included if they examined interventions designed to build implementation capacity collectively across researchers, practitioners, and/or practitioners-in-training (e.g., graduate students of professional programs such as nursing, social work, and other allied health fields)
  - Studies were included if they examined interventions designed to build implementation capacity among practitioners
  - Studies were included if they examined interventions designed to build implementation capacity among practitioners-in-training
- Studies that had relevance to academic institutions
  - Studies that met this criterion had one or more connections to an academic institution, faculty member(s), or academic medical center

#### Exclusion criteria:

- Studies that presented only theories, models, or frameworks for building implementation science capacity
- Studies that examined interventions designed to build implementation capacity among researchers only or researchers-in-training (e.g., research doctorate students)
- Studies that examined the implementation of a health-related practice, program, intervention, or innovation (e.g., smoking cessation program; cognitive behavioral therapy)
- Studies that only described capacity building interventions without reporting participant outcomes
- Review studies (e.g., systematic, scoping, rapid, narrative)
- Grey literature (e.g., government reports, dissertation/theses, conference proceedings, books)

In step 2 of our study selection process, these inclusion/exclusion criteria were applied to full-text articles by the same two review team members. Members discussed discrepancies on study inclusion until consensus was reached before proceeding to data extraction.

### Stage 4: Charting the data

Characteristics from each included study were extracted by the review team using an adapted data charting tool originally proposed by Arksey and O’Malley [19]. We chose to extract the following characteristics from each study given the relevance to our primary and secondary research questions: author and year, study location, target(s) of the capacity building intervention, core intervention components (described in detail in stage 5), intervention objective(s), and main outcomes. Reviewers met on a biweekly basis over the course of 2 months to reconcile discrepancies in data extraction.

### Stage 5: Collating, summarizing, and reporting the results

All included studies underwent numerical and open coding analyses. Numerical analysis was used to describe the types of studies included, the target audiences of capacity building interventions, and the number of core components integrated into each intervention. Two reviewers assessed the capacity building interventions described in each included study and used open coding to identify core intervention components. The reviewers drew language from Davis and D’Lima’s capacity building systematic review [7] as well as the Expert Recommendations for Implementing Change (ERIC) taxonomy [2] in order to differentiate intervention components. Table 1 lists the main core components extracted from each capacity building intervention and provides component descriptions. Reviewers repeated this open coding process to achieve uniform terminology for the types of outcomes measured to determine capacity building intervention effectiveness (Table 2).

**Table 1:** Description of core intervention components

Core intervention component	Description
Didactic activities	In-person or online coursework consisting of lectures, case studies; readings, and/or self-paced modules (e.g., slide-deck presentations)
Mentorship and expert consultation	Continued support that is initiated at the start of an implementation project and continued throughout project development and/or deployment; one-on-one or group meetings with leaders and faculty in implementation science and/or practice
Practical application activities	Small projects or field placements dedicated to implementing an evidence-based innovation under real-world circumstances
Knowledge sharing activities	Group meetings (online or in-person) that encourage networking, reflection, and the discussion of implementation experiences and reflections from implementation projects
Technical assistance	Email, phone, or website support for how to deliver an evidence-based innovation *or* an implementation strategy; specific reference to “technical assistance” or “technical coaching”

Descriptions informed by definitions drawn from Davis and D’Lima [7] and Powell et al. [2]

**Table 2:** Description of capacity building intervention outcomes

Outcome	Description
Knowledge attainment	Understanding and awareness of implementation models, factors and strategies influencing evidence-based practice implementation; evidence-based practice use
Increased ability to implement evidence	Perceived confidence, competence, or self-efficacy in initiating, leading, and/or participating in activities that facilitate evidence implementation
Productivity	Formation of implementation project teams; submitted grant proposals or manuscripts with an implementation focus; number of participants
Satisfaction	Perceived acceptability, appropriateness, or approval of capacity building intervention structure and content

## Results

**Fig. 2:**
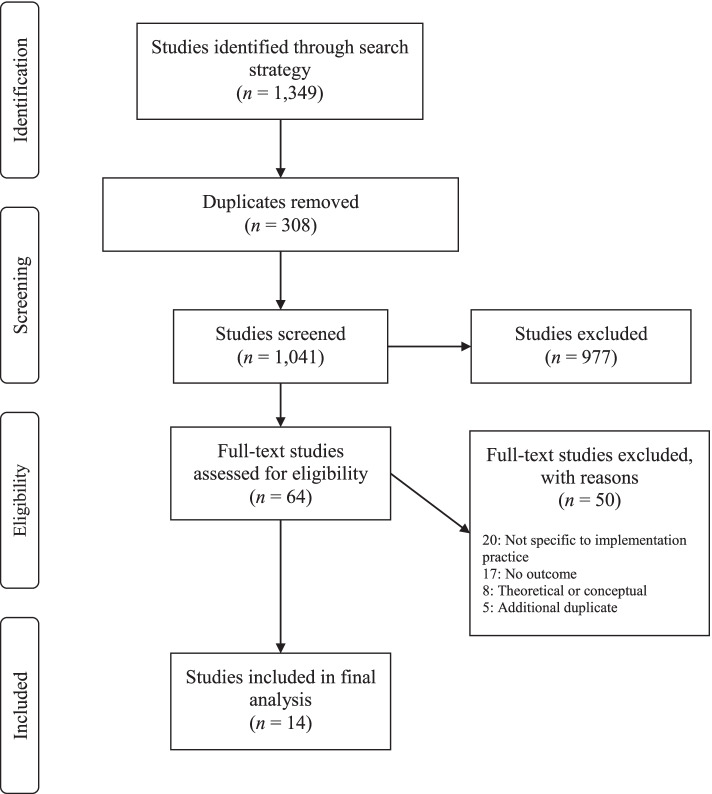
PRISMA flow diagram of study selection process

Our initial search strategy yielded 1349 studies that were entered into Covidence for screening. After title/abstract and full-text screening, a total of 14 studies were included in our final analysis (Fig. 2). Reviewers achieved a Cohen’s kappa of 0.49 during the review process, indicating a moderate level of agreement. Common reasons studies were excluded were lack of intervention outcome measurement, description of an intervention *not* unique to implementation practice capacity, and failure to clearly describe a specific capacity building intervention. The majority of included studies were descriptive in nature and none used experimental or quasi-experimental designs to evaluate the efficacy of the capacity building interventions.

### Targets of capacity building interventions

Researchers, practitioners, and practitioners-in-training (e.g., professional program graduate students) were all targets of implementation practice capacity building interventions. A total of eight interventions collectively targeted researchers, practitioners, and practitioners-in-training simultaneously [23–30], three interventions targeted practitioners only [31–33], and the remaining three targeted practitioners-in-training who represented the fields of social work [34], public health [35], and nursing [36].

#### Researchers, practitioners, and practitioners-in-training interventions

Studies that targeted researchers, practitioners, and practitioners-in-training were conducted in the USA [25–27], Canada [28, 30], South Africa [29], and Sweden [23], and jointly across the USA, Mexico, and India [24]. Each intervention consisted of a combination of core components with the most common interventions including didactic activities, mentorship and expert consultations, knowledge sharing activities, and practical application activities. As an example, from the University of Kentucky, the Value of Innovation to Implementation Program (VI^2^P) sought to build implementation capacity across its health system and six health professional colleges [25]. The VI^2^P was also designed to foster a local learning collaborative to facilitate implementation knowledge sharing, similar to the local implementation learning communities developed by the Colorado Research in Implementation Science Program (CRISP [26]) (Table 3).

**Table 3:** Characteristics of capacity building interventions for a combination of researchers, practitioners, and/or practitioners-in-training

	Country	Participants	Core components	Training objectives	Measurement	Outcomes
Carlfjord et al., 2017 [23]	Sweden	Researchers, allied health professionals	Didactic activities, knowledge sharing	To build understanding of implementation theory and practice in healthcare	Post-test survey	↑ knowledge; high satisfaction with program
Galaviz et al., 2016 [24]	USA, Mexico, and India	Mid-career professionals, researchers, and public health workers	Didactic activities, mentorship and expert consultation, practical activities	To build implementation capacity in LMICs	Pre-post survey at baseline and completion; total projects developed	↑ confidence in implementation research and leadership; development of 53 collaborative implementation projects
Li et al., 2019 [25]	USA	Researchers, practitioners, QI experts, community stakeholders	Mentorship and expert consultation, practical application activities, knowledge sharing activities	To foster a learning collaborative related to D&I capacity	Total implementation teams formed	Formation of 26 teams collaborating on implementation projects
Morrato et al., 2015 [26]	USA	Researchers, healthcare professionals/public health workers, graduate students	Didactic activities, mentorship and expert consultation, knowledge sharing activities	To adapt national D&I training programs and foster a local learning community	Pre-post survey at baseline, 1-week post, and 6-months post	↑ knowledge of D&I; ↑ development of D&I grants, papers, and projects
Norton 2014 [27]	USA	Researchers and public health students	Didactic activities, mentorship and expert consultation	To teach public health students and academic researchers about D&I	Post-test survey	↑ knowledge and confidence with D&I; high satisfaction with collaborative learning approach
Park et al., 2018 [28]	Canada	Healthcare professionals, researchers, managers	Didactic activities, mentorship and expert consultation, practical application	To build capacity for KT practice	Pre-post survey at baseline, 6-, 12-, 18-, and 24-months; interviews and focus groups	↑ self-efficacy, confidence, and knowledge in KT principles ↑ implementation of KT principles in practice
Ramaswamy et al., 2020 [29]	South Africa	Public health students and public health workers	Didactic activities, mentorship and expert consultation, practical application activities, knowledge sharing activities	To build implementation capacity in South Africa	Post-test survey; interviews	Reports of high satisfaction among participants
Straus et al., 2011 [30]	Canada	Researchers, healthcare professionals, graduate students	Didactic activities, mentorship and expert consultation, practical application activities, knowledge sharing activities	To build capacity in science and practice of KT	# of attendees	No data yet other than # of summer institute attendees (*n* = 90)

*LMICs* low- and middle-income countries, *QI* quality improvement

#### Practitioners

Three capacity building interventions targeted practitioners comprised of healthcare providers [31], clinical leaders and managers in behavioral health settings [32], and individuals in community-based service delivery [33] and were conducted in Canada [31] and the USA [32, 33]. All of these practitioner-only interventions incorporated didactic activities as a core intervention component with other common components including knowledge sharing activities and practical application exercises. For instance, intervention developers of the Training in Implementation Practice Leadership (TRIPLE) led activities to build implementation capacity among clinicians or administrators in behavioral health practice settings using the term “practice leader” to describe intervention targets. Core components of the TRIPLE intervention included the facilitation of knowledge sharing activities, didactic activities, consultations with implementation experts, practical application activities, and the provision of technical assistance [32] (Table 4).

**Table 4:** Characteristics of capacity building interventions for practitioners only

	Country	Participants	Core components	Training objectives	Measurement	Outcomes
Moore et al., 2018 [31]	Canada	Healthcare professionals	Didactic activities, mentorship and expert consultation, knowledge sharing activities	To provide training on the use of evidence and application of IS in healthcare	Pre-post survey at baseline, 3-, 6-, and 12-months; interviews	↑ knowledge and self-efficacy in implementation; high satisfaction
Proctor et al., 2019 [32]	USA	Clinical leaders and managers	Didactic activities, Knowledge sharing activities, mentorship and expert consultation, practical application activities, technical assistance	To promote leadership and organizational change and promote EBP implementation	Pre-post survey at baseline and completion	↑ competence High levels of program acceptability and appropriateness
Ramanadhan et al., 2017 [33]	USA	Community-based practitioners	Didactic activities, knowledge sharing activities, technical assistance	To promote EBP use in community-based organizations	Post-test survey and social network survey	↑ use of evidence in practice; reports of information sharing across CBO networks

*CBO* community-based organization

#### Practitioners-in-training

A total of three interventions, all delivered in the USA, described graduate-level academic work aimed at engaging students in implementation for professional careers in social work [34], public health [35], and nursing [36]. For instance, nurse practitioner students applied implementation concepts during their doctoral practice to gain experience leading efforts to implement evidence in practice [36]. Similarly, public health students engaged in opportunities to assess implementation determinants, strategies, and outcomes during in real-world contexts [33]. Masters of social work students developed implementation capacity by examining the delivery of a specific evidence-based practice at an assigned field site over a 16-week field rotation (Table 5).

**Table 5:** Characteristics of capacity building interventions for practitioners-in-training

	Country	Participants	Core components	Training objectives	Measurement	Outcomes
Bertram et al., 2018 [34]	USA	MSW graduate students	Mentorship and expert consultation, practical application activities, knowledge sharing activities	To enhance MSW students’ confidence implementing evidence-based social work practice	Qualitative review of student portfolios	↑ knowledge and confidence understanding program implementation
Ramaswamy et al., 2019 [35]	USA	MPH students	Didactic activities, knowledge sharing activities	To build implementation practice capacity among MPH students	End-of-semester evaluations; online discussions	High satisfaction, per student evaluation comments
Riner at al., 2015 [36]	USA	DNP students	Didactic activities, mentorship and expert consultation, practical application activities, knowledge sharing	To build implementation capacity of future nurse practitioners	Alumni surveys	↑ perceived ability to lead implementation efforts

*MSW* masters of social work, *MPH* masters of public health, *DNP* doctor of nursing practice

### Summary of capacity building intervention components

Our analysis yielded five common components of capacity building interventions across studies. These core components included didactic activities, mentorship and expert consultation, practical application activities, knowledge sharing activities, and technical assistance.

#### Didactic activities

Most often, capacity building interventions consisted of structured didactic activities that were delivered in-person, online, or in a hybrid format using a combination of lectures, readings, case studies, and self-paced modules. In-person didactic content was delivered in the form of university-level courses [23, 27, 34–36] or through workshop events led by implementation science experts [26, 28, 30–33]. Duration of in-person didactic workshops ranged from one and a half to 2 days. Hybrid didactic content was highly variable in structure and length. For instance, the PH-LEADER program lasted a total of 1 year consisting of a 2-month preparation period, a 3-week, in-person summer short course, and an in-country mentored project phase. Throughout the program, participants received didactic instruction from expert faculty as well as through recorded webinars [24]. Didactic content to build implementation capacity was also provided in an ongoing manner, such as through the Knowledge Translation Strategic Training Initiative in Canada [30]. The only didactic content delivered entirely online was structured in the form of a four-course series to build implementation capacity through the University of North Carolina Chapel Hill’s Gillings School of Global Public Health [35].

#### Mentorship and expert consultation

Across capacity building interventions, 11 included descriptions of formal mentorship and/or consultations from implementation experts to researchers, practitioners, or practitioners-in-training. Mentorship and expert consultation were provided to increase participants’ capacity to understand implementation principles and/or lead implementation-focused projects at their respective organizations. Mentorship and consultation activities occurred in settings such as academic institutions [25–27, 32, 34–36] and specialized institutes [28, 30, 31]. One global model of mentorship consisted of implementation faculty who provided training to field mentors. As part of this model, public health students and practitioners then completed implementation projects related to HIV/AIDS research in South Africa and received routine mentorship from their trained field mentors with a focus on implementation determinants, strategies, and outcomes [29].

#### Practical application activities

Interventions to build implementation capacity also included development and deployment of practical application activities. These activities allowed intervention participants to lead their own implementation projects in real-world contexts through pilot projects or evaluations of implementation determinants. Practical application activities were completed through graduate student field placements [34, 36], as well as small scale implementation projects [24, 28, 30, 32, 35]. In the academic medical center setting, Li et al. [23] facilitated practical application of implementation principles by convening an informal network of implementation researchers, practitioners, students, quality improvement experts, and community stakeholders. Individuals within this network formed implementation teams who submitted grant applications (funded through the University of Kentucky) to complete implementation-related projects by partnering with medical center affiliates. Of the 26 teams who submitted applications, four projects were funded as of 2019, allowing teams to gain practical experience conducting projects informed by implementation methodologies.

#### Knowledge sharing activities

Knowledge sharing took the form of small group reflections and exercises, expert panel discussions, breakout activity sessions, and the development of learning collaboratives [25, 26, 29, 30, 32, 33, 35, 36] Among practitioners-in-training, as one example, social work graduate students examined the implementation of evidence-based practices over a 16-week field placement, and students completed weekly field portfolios that described the process of implementing specific evidence-based practices. Weekly cohort seminars allowed students to share content from their portfolios and their experiences implementing evidence within their field placement sites [34]. For practitioners involved in the Practicing Knowledge Translation program, participants were encouraged to engage in small group discussions and share their progress towards completing their implementation and learning goals [31].

#### Technical assistance

Capacity building interventions also provided technical assistance to facilitate the development of implementation projects and grant proposals. Two studies described using this type of assistance—referred to explicitly as technical assistance or technical support—which included building implementation support networks, identifying appropriate implementation projects to deploy in the community setting, and facilitating implementation training opportunities for participants [32, 33]. Specifically, the TRIPLE program aimed to build implementation capacity among behavioral health organizations and provided technical support to assist mid-level organization leaders in their implementation of evidence-based practices at their own agencies. Technical assistance (e.g., developing project activities; planning for outcome measurement) was offered by TRIPLE faculty who provided coaching on the development of implementation projects, the theories, models, and frameworks that inform implementation, strategies for implementing change, and methods for conducting organizational change evaluations [32].

### Outcomes of capacity building interventions

All interventions included in this review described favorable outcomes. Outcomes were categorized into the following groups that are described in further detail below: knowledge attainment, increased perceived ability to implement evidence, productivity, and satisfaction.

#### Knowledge attainment

Six studies in the review explicitly gathered data on overall knowledge of implementation [23, 26, 28, 31, 34, 37], defined as the understanding and awareness of implementation models, factors, and strategies influencing evidence use. Moore and colleagues [31], for example, conducted a longitudinal study of practitioners’ knowledge and application of implementation principles at intervals over a year; all subjects showed a significant increase of knowledge, which Moore et al. associated with improved application of implementation methodologies to local projects. Bertram and colleagues [34] built implementation capacity through graduate coursework and found that the course components led to increased knowledge and understanding of implementation models, factors influencing program implementation, and implementation interventions.

#### Increased perceived ability to implement evidence

A total of seven studies had outcomes specifically targeting participants’ ability to implement evidence into practice, as measured through self-efficacy, confidence, and competence in evidence implementation [24, 28, 31–34, 36]. Of these studies, only four measured self-efficacy or confidence at multiple time points [24, 28, 31, 32], whereas the two academic courses targeting social work [34] and nursing [36] students used student feedback in the form of narrative course evaluation comments to assess outcomes. Tools used at baseline and follow-up included the Evidence-Based Practice Confidence Scale [38], a 3-item tool measuring intentions to use evidence [39], the Implementation Leadership Scale [40], the Implementation Climate Scale [41], and the Organizational Readiness for Implementing Change scale [42] as well as original surveys that were created by capacity building intervention developers.

#### Productivity

Productivity was not clearly defined as an outcome in the included studies but represents the number of researchers, practitioners, and practitioners-in-training whose implementation-related productivity changed as a result of participating in capacity building interventions. Measures of productivity were often reported in lieu of other objective outcomes, such as self-efficacy and knowledge. Examples of productivity included the development of over 50 collaborative implementation projects in low- and middle-income countries after public health professionals and researchers participated in a global capacity building intervention [24]. Similarly, Li et al.’s [25] implementation network facilitated the formation of 26 teams who submitted internal grant applications to conduct implementation studies with academic medical center partners. Studies that did not measure team or project formation reported productivity in the form of the number of participants reached [30] and the amount of implementation knowledge shared across organizations [33].

#### Satisfaction

Satisfaction with capacity building interventions was measured through both qualitative and quantitative methods. Student evaluation reports indicated high levels of satisfaction with online implementation coursework [23, 35], and program evaluations indicated favorable satisfaction with practical application activities [29] and collaborative learning exercises [27]. Proctor et al. [32] was the only study that quantitatively evaluated participants’ perceptions of their TRIPLE program by using the Training Acceptability and Appropriateness Scale which was administered at the post-test time point only. The Training Acceptability and Appropriateness Scale is an unpublished, 14-item measure with high internal consistency that assesses the extent to which a training intervention is acceptable, feasible, and appropriate for participants based on their self-perceived needs.

## Discussion

As the field of implementation science has grown over the past 15 years, new insights about the complexities and strategies for moving effective interventions into routine care settings highlight a range of implementation skills and capacities for practitioner and policy leaders. Academic institutions building capacity for implementation scholarship are also well positioned to build capacity among those in real world health and human service settings, although how practitioners are included and trained in these initiatives is unclear [7]. This scoping review identified 14 studies of implementation capacity building interventions that included practitioner stakeholders and also had ties to academic institutions. Results demonstrate how initiatives have targeted a range of stakeholders including practice leaders and students, often using traditional didactic or mentored training approaches with a goal of improving implementation knowledge, self-efficacy, skills, and capacities. Institution-wide efforts to build connections among implementation teams of practitioners and researchers were rare but might offer important opportunities to build capacity for implementation research and practice in concert. These results have direct implications for how other academic institutions design implementation capacity building interventions.

It is important to note that the capacity building interventions identified in this review target an array of practitioner and student/trainee stakeholders by pairing them with an expert in implementation science in a variety of settings. Additionally, the capacity building interventions reported building student or practice leader capacity via expert-led initiatives, which tended to be formal and structured programs. While these were generally effective, few programs described leveraging existing university/practice partnerships (perhaps even within their own healthcare systems) to create collaborative learning and network building to support implementation science approaches across practice settings. This is an important gap to highlight, as expanding current partnerships to build capacity for implementation science and application is a feasible approach with potential for immense gains [43].

### Intervention components

Didactic activities, either online or in person, as well as expert consultation, continue to be used most often in implementation practice capacity building interventions. Didactic coursework models typically rely upon centralized implementation experts who are responsible for training and supporting participants. Other larger initiatives are often housed within an institution or system and focus on building networks and sharing knowledge across teams and stakeholders. Within this larger model, expertise is often distributed under the assumption that capacity is built by connecting teams and stakeholders to share their knowledge and expertise across settings. Structured learning activities provide opportunities to gain important skills while also encouraging network building among participants. These intervention components are similar to other collaborative learning models (e.g., [44]) suggesting their promise for developing implementation practice capacity among diverse stakeholders.

### Outcomes

A number of outcomes were reported across studies included in our review. Outcomes included knowledge of implementation and/or its application, self-efficacy and confidence implementing evidence in practice, collaborations with researchers and practitioners, and participant satisfaction. Productivity was reported mostly in numbers of projects and teams created. While most programs reported positive gains across outcomes measures, there was wide heterogeneity in methods of measurement and time points for evaluation. Several interventions reported on immediate educational outcomes or included a singular point in time for follow-up measures. Because many initiatives are often designed with the long-term goal of improving implementation in real-world settings, additional implementation outcomes that might be especially relevant to practitioners may include adoption, fidelity, penetration, and sustainability [45]. Future program descriptions and evaluations should consider use of these additional outcomes measures to better detect degree of impact and diffusion of capacity building interventions over time and across a variety of practice settings.

### Limitations

There are inherent limitations to this scoping review summary. The primary limitation is that findings are limited to only published studies of academic/practice-based capacity building interventions. As such, content does not include current or ongoing implementation practice programming for practitioners that is delivered in non-academic settings, has yet to be evaluated, or has yet to be published in the academic literature. Another limitation to the interventions described in our review was that nearly all included reports were descriptive, which is important for informing designs in other settings, but does not allow for inferences about their effectiveness. Future work in this area would benefit from more rigorous designs for examining the educational and implementation outcomes of capacity building interventions. Lastly, there was substantial variation in how capacity building interventions components (e.g., didactic activities) and outcomes were described. While our open coding analysis with two reviewers aimed to address this limitation, it should be noted that the definitions of intervention components and outcomes were established by the review team rather than by the original capacity building intervention developers.

### Implications and future directions

The field of implementation science has generated important knowledge to streamline integration of research evidence into a multitude of practice settings. After over 15 years of focused research advancements in this field, additional attention is now needed to integrate this knowledge into educational programs in health and allied healthcare fields to prepare future leaders and practitioners in the practice of science-based implementation. Simultaneously, programming for building capacity should also target existing practice leaders who have potential and reach to integrate skills into their agencies and teams. Training non-provider leaders can also bring needed support and resources to busy practice settings. Notably, none of the capacity building interventions in this review identified targeted policy makers, despite their role in generating policies and regulations to support the adoption and sustainability of evidence-based interventions. Working with policy makers to expand their implementation knowledge and skills for using research evidence might be an area for innovative partnerships that has potential to lead to needed studies of policy implementation strategies, and changes to policy practices that support implementation [46, 47]. Building this type of implementation capacity across current and future academic, practice, and policy settings will prevent development of a secondary implementation research to practice gap.

## Conclusion

While D&I trainings for researchers have proliferated in recent years, it is unclear whether and how comparable parallel efforts have targeted communities of practitioners to integrate insights from implementation science about planning, strategies, adaptation, or sustainability of best practices. For instance, the field lacks evidence or descriptions of how implementation science insights have been integrated into professional graduate training programs, continuing education workshops, and other professional development opportunities for practice leaders. It is also unclear whether the majority of capacity building interventions train community practitioners and researchers to collaborate in service of generating new evidence about strategies for integrating effective interventions within local contexts and the degree of diffusion of those findings across similar networks. Ultimately, the goal of any capacity building initiative is to yield long-term, sustainable impact over time and across settings and teams. This includes increasing the number of evidence-based interventions that are implemented and evaluated, reducing the time it takes for integration into real-world practice. As participants in capacity building interventions build knowledge, skills, and competencies, program initiatives may need to evolve to incorporate more advanced or sustained components and supports. As such, long term evaluations of these programs over time is necessary.

## Supplementary Information

**Additional file 1.**

## Data Availability

Not applicable as extracted data from study articles are included in Tables 1, 2, and 3
